# Advancing Global Health Surveillance of Mycotoxin Exposures using Minimally Invasive Sampling Techniques: A State-of-the-Science Review

**DOI:** 10.1021/acs.est.3c04981

**Published:** 2024-02-14

**Authors:** Tyler A. Jacobson, Yeunook Bae, Jasdeep S. Kler, Ramsunder Iyer, Runze Zhang, Nathan D. Montgomery, Denise Nunes, Joachim D. Pleil, William E. Funk

**Affiliations:** ‡ Department of Preventive Medicine, Northwestern University Feinberg School of Medicine, Chicago, Illinois 60611, United States; § University of Michigan Medical School, Ann Arbor, Michigan 48109, United States; ⊥ Galter Health Sciences Library, Northwestern University Feinberg School of Medicine, Chicago, Illinois 60611, United States; || Department of Environmental Sciences and Engineering, Gillings School of Public Health, University of North Carolina, Chapel Hill, North Carolina 27599, United States

**Keywords:** dried blood spots, dried serum spots, biomonitoring, mycotoxins, ochratoxins, aflatoxins, fumonisins, volumetric tip microsampling

## Abstract

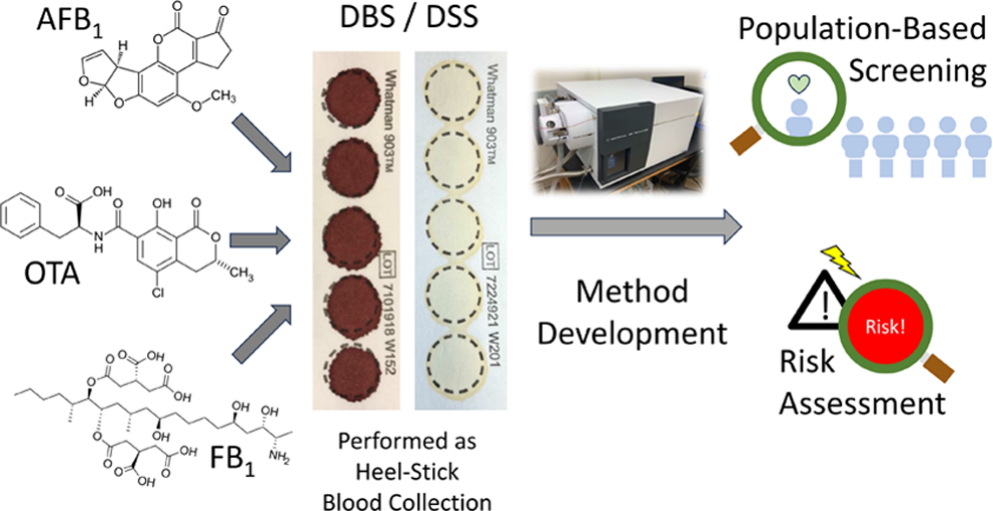

Mycotoxins are a heterogeneous group of toxins produced by fungi that can grow in staple crops (e.g., maize, cereals), resulting in health risks due to widespread exposure from human consumption and inhalation. Dried blood spot (DBS), dried serum spot (DSS), and volumetric tip microsampling (VTS) assays were developed and validated for several important mycotoxins. This review summarizes studies that have developed these assays to monitor mycotoxin exposures in human biological samples and highlights future directions to facilitate minimally invasive sampling techniques as global public health tools. A systematic search of PubMed (MEDLINE), Embase (Elsevier), and CINAHL (EBSCO) was conducted. Key assay performance metrics were extracted to provide a critical review of the available methods. This search identified 11 published reports related to measuring mycotoxins (ochratoxins, aflatoxins, and fumonisins) using DBS/DSS and VTS assays. Multimycotoxin assays adapted for DBS/DSS and VTS have undergone sufficient laboratory validation for applications in large-scale population health and human biomonitoring studies. Future work should expand the number of mycotoxins that can be measured in multimycotoxin assays, continue to improve multimycotoxin assay sensitivities of several biomarkers with low detection rates, and validate multimycotoxin assays across diverse populations with varying exposure levels. Validated low-cost and ultrasensitive minimally invasive sampling methods should be deployed in human biomonitoring and public health surveillance studies to guide policy interventions to reduce inequities in global mycotoxin exposures.

## Introduction

Despite significant progress in our understanding of the health effects of mycotoxins over the past 50 years, human exposure to mycotoxins has remained an under-recognized global health issue.^1−3^ Significant inequities exist in exposures to mycotoxins globally with elevated exposures occurring in many low- and middle-income countries (LMICs) with poor legislation and regulatory mechanisms to monitor the food supply chain.^1,3−6^ Mycotoxins are secondary metabolites produced by fungi and can contaminate food, including maize, cereals, groundnuts, and tree nuts, resulting in widespread exposure due to direct (e.g., consumption of grain-derived foods) and indirect (e.g., milk or meat from animals with feeds contaminated with mycotoxins) human consumption.^7,8^ Animal exposure to mycotoxins and carryover effects in humans is succinctly reviewed by Nji et al.^8^ Human exposure to mycotoxins may also occur in the environment via inhalation.^7^

Mycotoxins may adversely affect human health and consequently should be incorporated into human biomonitoring (HBM)^9^ and exposomics studies.^10^ Among the mycotoxins, aflatoxins, fumonisins, ochratoxins, deoxynivalenol (DON), and zearalenone (ZEA) pose the greatest threats to global health.^3,11^ The European Human Biomonitoring initiative (HBM4 EU) recently labeled aflatoxin B1 (AFB_1_), DON, and fumonisin B1 (FB_1_) as “prioritized chemicals of concern”^12^ and there is growing interest in monitoring and reducing mycotoxin exposure levels globally.^4^ While much progress has been made in measuring aflatoxin exposure biomarkers to understand disease etiology and evaluate public health interventions to reduce exposures,^1,2^ there remains a paucity of high-quality longitudinal epidemiologic data on human exposures to most mycotoxins and associated health risks, especially in children.^13,14^ Although much can be attributed to a lack of proper surveillance in many parts of the world,^3^ the surveillance mechanisms which exist primarily involve measurements of food contamination (i.e., food sampling) rather than measurements of human exposures (i.e., biological sampling).

Populations in many LMICs face higher chronic exposures to mycotoxins due to climate and storage conditions that are conducive to fungal growth and mycotoxin production (e.g., high heat and humidity), exacerbated by poor or nonexistent food surveillance systems.^3,15^ Rural subsistence farming communities face the greatest exposure risks because many of these communities both produce and consume their own crops without regulatory oversight.^3,8^ In addition, climate change is expected to increase human exposure to mycotoxins in many geographic regions,^7^ including Europe and other regions that may experience more extreme weather events, such as heat waves and droughts.^16^ More research is needed to identify the effects of climate change on exposures to mycotoxins in geographic areas where exposures are endemic,^8,17,18^ including regions in Africa and Asia where concerning elevations in levels of aflatoxin exposure biomarkers have been reported.^3^

While aflatoxins are recognized to be highly hepatocarcinogenic in humans, especially in those with chronic hepatitis B virus (HBV) infection,^1,3^ other possible health effects from aflatoxins and other mycotoxins have been less well studied. Epidemiologic studies have investigated the associations between dietary mycotoxin exposure (mostly aflatoxins) and child growth impairment, immune system effects, morbidity, and mortality. However, the overall quality of the evidence is low, and the results are inconclusive.^19^ A variety of biological mechanisms have been proposed by which mycotoxins may exert effects on human health,^3,20,21^ including on the gut microbiota.^22^ Mycotoxin exposure may also increase the risk for adverse fetal and maternal outcomes.^23^ Aflatoxins, for example, can cross the placental barrier and exert effects *in utero.*(3) Carefully designed prospective cohort studies and cluster randomized controlled trials^19,24^ are needed to elucidate health risks attributable to exposure to mycotoxins and evaluate interventions which can reduce exposures.^3^

Dried blood spot (DBS) sampling is an emerging tool for public health and environmental epidemiology, and has particular advantages in low-resource settings and in studies involving infants and children.^25^ DBSs are drops of whole blood from a minimally invasive finger- or heel-prick, collected on specially designed filter paper (e.g., Whatman 903).^26,27^ We recently reviewed the state of the science of using DBS sampling for measuring exposures to environmental tobacco smoke, trace elements (lead, mercury, cadmium, and arsenic), several important persistent organic pollutants (e.g., per- and polyfluoroalkyl substances; PFAS), and endocrine disrupting chemicals.^25^ The number of developed and validated DBS assays for measuring environmental exposure biomarkers continues to grow,^25,28,29^ with many recent applications in larger-scale field studies conducted in low-resource settings.^25^ Volumetric tip microsampling (VTS) is another minimally invasive sampling technique where a fixed volume of blood (∼10.4 μL) is absorbed on a tip, therefore potentially overcoming drawbacks of blood volume variations (homogeneity issues) and hematocrit effects with DBS/DSS punching methods.^30,31^

DBS sampling and VTS are suitable for estimating long-term and chronic exposures to mycotoxins because covalently bound mycotoxin exposure biomarkers, such as AFB_1_-lysine^2^ and Ochratoxin A (OTA),^32,33^ have long retention times in human blood (e.g., half-life: ∼35 days for OTA)^9,32,34^ due to their high binding to plasma proteins (e.g., human serum albumin).^2,9,35,36^ This can help to overcome limitations of using single spot measurements with biomarkers with short biological half-lives (e.g., urinary metabolites) in cross sectional studies for determining the etiologies of multifactorial chronic diseases.^37−39^ For single spot measurements, intraclass correlation coefficients (ICCs) can be reported to assist in interpreting the reliability of the exposure biomarker.^38,40^ ICCs are determined from repeated measurements from the same individual over various time periods, compared to variations between individuals.^40^ Minimally invasive sampling methods can facilitate repeated measurements of mycotoxin exposure biomarkers in longitudinal cohort studies, which can play a key role in establishing ICCs for various mycotoxin biomarkers in humans.

This review summarizes assays using minimally invasive sampling techniques (DBS/DSS and VTS) for estimating exposures to mycotoxins in human blood and suggests the next steps, including lab and field- validation, required to incorporate minimally invasive sampling methods into large-scale epidemiologic studies and global public health interventions.

## Methods

A systematic search of the literature (PubMed, Embase, and CINAHL) was conducted in March 2022. Details on this systematic search, including original search terms, were reported previously.^25^ The search strategy was developed collaboratively by one of the lead authors (T.A.J.) and a health sciences research librarian at Northwestern University (Chicago, IL, USA) (D.N.). The search was designed to identify all reports of environmental exposure biomarkers in human DBS samples.^25^

### Inclusion criteria

- Developed, validated, and/or applied methods to measure mycotoxin exposure biomarkers. We included two reports which measured a biomarker of effect,^41,42^ since this biomarker is specific to environmental exposure to fumonisins.
- Used a minimally invasive sampling technique, such as dried blood spot (DBS), dried serum spot (DSS), or volumetric tip microsampling (VTS)/volumetric absorptive microsampling (VAMS).
- Assays were developed for human blood samples (not animal samples).

We updated our systematic search in October 2023 by adapting our original search terms to PubMed (more details in Supporting Information, S1). In addition, we reviewed the reference lists of included studies and used Google Scholar and Web of Science to follow citation trails of included reports to identify new studies published since our previous search (March 2022). With our updated search, we identified a total of 11 reports which described components of development, validation, and/or application of DBS/DSS or VTS assays for measuring mycotoxins in human blood samples (more details in S1). Key metrics of assay performance were extracted by the lead author (Y.B.) and spot checked (T.A.J., J.S.K., R.Z.). These metrics were organized into Table 1 (descriptive study data) and Table 2 (study outcomes data).

**Table 1:** Experimental Conditions of Each DBS Method Development Study^a^

Study	Target compounds	Exposure (biomarker)	Instrument	Punch size and/or sample volume^d^	Reconstituted volume for LC analyses	Reconstituted quantity^f^ (mm/μL)	Sample type and size: case/control	Locations of sample collected	Published country
Cramer et al.^34^	OTA	OTA	HPLC-MS/MS	Spot corresponding to **100 μL** DBS (∼ **20 mm**)	100 μL^e^ (W:M:F = 60:40:0.1)	0.2	**50 participants**:	Műnster, Germany	Germany
		2’R-OTA					29 male, 21 female		
Osteresch et al.^48^		OTA	HPLC-MS/MS	• **8.8 mm** punch (**∼20 μL** of blood) •		8.8 mm punch: 0.088	34 coffee drinkers, 16 noncoffee drinkers		
		2’R-OTA		Spot corresponding to **100 μL** DBS (∼**20 mm**)		100 μL DBS: 0.2	Age: 18 and >60 years.		
Osteresch et al.^46^	OTA, FB_1_	27 mycotoxins and mycotoxin metabolites^b^	HPLC-MS/MS	Spot corresponding to **100 μL** DBS or DSS (∼**20 mm**)	Volume not specified^e^ (W:A:AC = 95:5:0.1)	-	**50 German cohorts**: for applying developed 27-mycotoxins-method		Germany/Portugal
Xue et al.^35^	AFB_1_	AFB_1_ (AFB_1_-lysine)	HPLC-FDS^c^	• **12.7 mm** punch (**50 μL** whole blood)	150 μL (25% MeOH)	8.47 × 10^–2^	**36 participants** (Kenyan mother and children): low, medium, and high dietary AFB_1_ exposure (no case/control)	Kenya	United States
			LC-MS/MS						
Riley et al.^41^	FB_1_	Sa-1-P	HPLC-MS/MS	• **8 mm** punch (**∼17 μL** whole blood)	Volume not specified	-	Volunteers who consumed maize-based foods^g^(*n* = 186)	• Athens, Georgia, USA; Chimaltenango and Escuintla, Guatemala	
		So-1-P							
Riley et al.^42^	FB_1_	Sa-1-P	HPLC-MS	• **6-** or **8 mm** punch (**∼17 μL** whole blood)	Volume not specified	-	Large field study: 1240 women	Guatemala	United States
							low exposure: *n* = 841		
							high exposure: *n* = 399		
							Confirmatory field study: 299 women		
		So-1-P					low exposure: *n* = 100		
							high exposure: *n* = 199		
Renaud et al.^47^	AFB_1_	AFB_1_ (AFB_1_-lysine)	LC-MS/MS HPLC-FLD^c^ ELISA	Not specified^h^	380 μL^i^	-	Not specified	jN/A	Canada
Vidal et al.^30^	OTA, FB_1_, AFB_1_	24 mycotoxins	UPLC-MS/MS^k^	10.4 μL^l^	50 μL	0.126	mN/A		

a Aflatoxin B_1_ (AFB_1_), Enniatin B (EnB), fumonisin B_1_ (FB_1_), ochratoxin A (OTA), sphinganine 1-phosphate (Sa-1-P), sphingosine 1-phosphate (So-1-P), and 2’R- ochratoxin A (2’R-OTA).

b The list of these 27 mycotoxins and mycotoxin metabolites are described in Table 3.

c FDS: fluorescence detection system; FLD: fluorescence detector.

d Blood volume estimates for each disc size were derived using blood applied to a blank filter paper spot.

e W: water; M: methanol; F: formic acid; A: acetonitrile, AC: acetic acid.

f Reconstituted quantity (mm/μL) = disk area (mm)/reconstituted volume (μL).

g From cohorts in Athens, Georgia: *n* = 10; From cohorts in Guatemala: *n* = 76 women and 100 men.

h The DBS paper was carefully excised and cut into 8 pieces with a scalpel.

i 111.5 μL PBS buffer + 40 μL H_2_O + 20 μL Pronase + 8.5 μL 2nd internal standard +200 μL MeOH = 380 μL

j AFB_1_-serum albumin (SA) reference material was used.

k Ultraperformance liquid chromatography - tandem mass spectrometry.

l Corresponds to 6.3 mm of DBS punch.

m Blood was purchased instead of collected from human participants.

**Table 2:** Summary of Key Findings of Each DBS Method Development Study

Study	Exposure (biomarker)		Precision (RSD^k^)	Reliability^g^	Recovery rate	Accuracy	SensitivityLOQ (unit: ng/mL unless otherwise indicated)	Stability	Note
Cramer et al.^34^	OTA		OTA: 3.1%^k^	RSD for OTA: 7.3% • 2’R-OTA: 7.5%	100 μL DBS OTA: 101–105%; 2’R-OTA: 99–105%	Both OTA and 2’R-OTA: r^2^ >0.99	2’R-OTA: 0.021	4 °C in the dark: analytes were stable for >4 weeks	-
	2’R-OTA		2’R-OTA: Not measured^a^		18.7 μL DBS (8.8 mm disk) OTA: 101–105%; 2’R-OTA: 99–105%				
Osteresch et al.^48^	OTA			Spiked whole DBS (8.8 mm) (0.05–1.00 ng/mL) OTA and 2’R-OTA: r^2^ > 0.99.		OTA: r^2^ = 0.93; 2’R-OTA: *r*^2^ = 0.91 (venous-blood-spikedvs finger-prick DBS)	OTA, 2’R-OTA: 0.021		• Used DBS assay developed by Cramer et al.^34^ • Hematocrit effects negligible
	2’R-OTA								
Osteresch et al.^35,46^	27 mycotoxins and metabolites	26 mycotoxins^e^	0.5–13.8%^c^	8.4–21.6% RSD depending on mycotoxins	• 24 out of 27 mycotoxins: 80–120%	27 analytes: *r*^2^ > 0.99^d^	0.005–5.0 depending on analyte	**AFB**_**1**_**recovery rates**: 20 °C, > 84% (1 week); 44% (5 weeks); 17% (10 weeks); 4 °C, app. 80% (24 weeks); −18 °C, > 94% (24 weeks).	Biomonitoring: German cohort (*n* = 50) HPLC-MS/MS approach applied for other studies: (43)^,^(44)^,^(45)
					DON: 77%		OTA, 2’R-OTA: 0.05		
		FB_1_	-	-	DBS: 97% •	-	2.5	20 °C: 55% (5 weeks), 37% (10 weeks). −20 °C, > 95% (24 weeks).	
					DSS: 61%				
Xue et al.^35^	AFB_1_-lysine		<7% for AFB_1_-lysine	<6% RSD for AFB_1_-lysine	68–72% for AFB_1_-lysine	*r* = 0.78 (*p* < 0.0001). (DBS vs Serum)	0.4 pg/mg albumin	4 °C storage : stable for 12 months	Field study: cohorts from Kenya (*n* = 36)
Riley et al.^41^	Sa-1-P		So: 10%^b^	No significant increase for both Sa-1-P and So-1-P^c^	So: 73%	• Sa-1-P: r^2^ = 0.96	Both So 1-P and Sa 1-P: 0.8 pmol from 8 mm spot	Stored at −20 °C: stable for 170 days	Pilot study ) in Guatemala ( *n* = 176)
			Sa: 6%^b^		Sa: 72%				
	So-1-P		So 1-P: 5%^b^		So-1-P: 61%	So-1-P: *r*^2^ = 0.99			
			Sa 1-P: 4%^b^		Sa-1-P: 74%				
Riley et al.^42^	Sa-1-P		-	-	-	-	-	-	Women in Guatemala Large field study (*n* = 1240) Confirmatory field study:(*n* = 299)
	So-1-P								
Renaud et al.^47^	AFB_1_-lysine		VTS: 4.8–22.6%	-	VTS: 81.0–114%	*r*^2^ = 0.99 (DBS vs VTS)	-	-	-
			DBS: 3.6.–19.0%		DBS: 54.9.–92.9%				
Vidal et al.^30^	OTA		j	j	• OTA: 85–91%^f^	OTA and AFB_1_ had similar detection rates for VTS and whole blood.	OTA: 0.36	**After 7 days*****At***20–23**°C** (***Room Temp.***)^i^: OTA: 91–117%, AFB_1_: 94–129%, FB_1_: 78–106%. ***At 4 °C*** : OTA: 88–100%, AFB_1_: 88–101%, FB_1_: 77–103%	-
	AFB_1_				AFB_1_: 88–111%^f^	**Average ± SD**^h^**(ng/mL)*****OTA***: VTS: 0.56 ± 0.12, whole blood: 0.42 ± 0.18.	AFB_1_: 0.07	**After 21 Days,*****At***20–23**°C** (***Room Temp.***): OTA: 103–109%, AFB_1_: 86–119%, FB_1_: 81–92%. ***At 4 °C***: OTA: 90–119%, AFB_1_: 92–121%, FB_1_: 68–96%	
	FB_1_				FB_1_: 85–104%^f^	***AFB***_***1***_: VTS: 0.10 ± 0.06., whole blood: 0.09 ± 0.08	FB_1_: 3.09		

a Obtained from the sample with the lowest OTA concentration.

b Obtained from cards spiked with a standard mixture.

c From human volunteers (*n* = 7) consuming maize-based foods for 3 days.

d Specific *r*^2^ values of each analytes are illustrated in Table 2 in Osteresch et al.^46^

e FB_1_ excluded.

f Recovery rates varied depending on spiked concentrations.

g Reproducibility (Interassay CV).

h Standard deviation.

i Stability varied depending on spiked concentrations.

j Even though Vidal et al.^8^ calculated precision and reliability, the authors did not report the specific values in their report.

k RSD: relative standard deviation.

## Overview

A total of 11 reports have described and/or applied validated methods for measuring mycotoxins in DBS/DSS samples and VTS. Eight reports were primarily methods development and validation (Table 1), while 3 reports were primarily application of previously validated assays.^43−45^ Three reports developed and/or validated DBS/DSS methods for detecting biomarkers of exposure to aflatoxins,^35,46,47^ three reports developed methods for ochratoxins,^34,46,48^ two reports developed methods for detecting fumonisins mechanism-based biomarkers,^41,42^ and one report described methods for detecting fumonisins directly in DBS/DSS samples.^46^ Multimycotoxin asssays have been described for both DBS/DSS^46^ and VTS.^30^ No studies utilized newborn DBS samples. DBS measurements were compared to matched venous blood values in two studies.^35,48^

Among the studies which were primarily application of previously developed assays, one study collected DSS samples from waste management workers in Portugal,^43^ one study used DSS samples in a relatively large (*n* = 1105) survey of school children in Sweden,^45^ and one study used DSS samples to compare mycotoxin exposure among vegans and omnivores using a cross sectional study design.^44^ No application studies collected DBS/DSS samples in the field, i.e., all three studies spotted DSS samples from venous blood samples.^43−45^ Three larger-scale field studies collected DBS samples in Guatemala.^41,42^ One field study was conducted using previously collected DBS samples from a longitudinal cohort study in Kenya^35^ and one study used DBS samples collected in a German cohort.^34,46,48^ In total, three reports were from Germany,^34,44,48^ two reports were from Germany/Portugal,^43,46^ one report was from Belgium,^30^ one report was from Sweden,^45^ three reports were from the United States,^35,41,42^ and one report was from Canada.^47^ Additionally, the extraction of mycotoxin biomarkers from DBS/DSS and VTS generally includes two stages: (1) sonication of the sample with a water/acetonitrile mixture and (2) digestion with protonase. Specific key extraction procedures conducted by each study are summarized in Section S2 of the Supporting Information.

## Aflatoxins

### Background

Aflatoxins can contaminate groundnut- and corn-based foods and is of particular concern in LMICs, including many countries in sub-Saharan Africa and Southeast Asia.^2,49−52^ Exposure to aflatoxins is a well-recognized risk factor for liver cancer and may also increase the risk for childhood stunting.^3,49−51^ AFB_1_ is classified as a Group 1A carcinogen by the International Agency for Research on Cancer (IARC).^53^ A recent study reported an increase in the serum AFB_1_-lysine adduct levels from 2004 to 2014 from 2.35 to 4.34 pg/mg albumin among two populations in Texas, United States.^54^ Therefore, more HBM studies are needed, especially among populations with expected increases in exposure to aflatoxins with global climate change.^16^

### Methods

Xue et al. (2016) validated previously developed methods^55,56^ for measuring aflatoxin B1 (AFB_1_) lysine adducts in DBS samples using a high-performance liquid chromatography (HPLC)-fluorescence detection system (FDS). The method for detecting and quantifying AFB_1_-lysine adducts in DBS was further confirmed and validated using LC-MS/MS.^35^ DBS methods were developed and validated in rats and then applied to quantify AFB_1_ lysine adduct levels in DBS samples (*n* = 36) from mothers and children in a previous longitudinal study in Kenya.^35^ Recovery rates of spiked rat DBS samples were close to 100% regardless of spotted blood volumes (20, 40, or 60 μL of blood).^35^ The LOD was determined to be 10 pg/mL of extract of 0.2 pg/mg of albumin, and the assay produced precise and reliable results. AFB_1_-lysine adducts showed a strong dose–response relationship with administered doses of AFB_1_ in the animal model.

Average AFB_1_-lysine adduct levels in the human DBS samples was 11.88 pg/mg albumin.^35^ Human DBS and serum sample AFB_1_-lysine adduct levels had a Pearson correlation coefficient of 0.784 (*p* < 0.0001).^35^ While detection rates were 100% in the high exposure group (*n* = 12, median adduct concentration = 136 pg/mg albumin), detection rates were around 50% in both the low (*n* = 12, median adduct concentration = 4 pg/mg albumin) and medium exposure groups (*n* = 12, median adduct concentration = 12 pg/mg albumin).^35^ Therefore, future work may seek to further lower the detection limits of this assay. Adducts in DBS samples were stable at 6 and 12 months when refrigerated at 4 °C in bags with desiccant.^35^

Osteresch et al. (2017) developed and applied an assay to measure a total of 27 important mycotoxins and metabolites using HPLC-MS/MS, including the direct measurement of several aflatoxins (Aflatoxin B_1_; Aflatoxin B_2_; Aflatoxin G_1_; Aflatoxin G_2_; Aflatoxin M_1_).^46^ The scheduled multiple reaction monitoring (sMRM) parameters for HPLC-MS/MS of all 27 mycotoxin analytes are reported.^46^ DBS and DSS samples were spiked with 100 μL of blood or serum from healthy volunteers from Germany, respectively. Using spiked DBS/DSS samples, recovery rates were high (>81%, with most close to 100%) for all target aflatoxins in both DBS and DSS samples.^46^ Detection limits, including comparison to HPLC-MS/MS analyses of whole blood (DBS) and plasma (DSS) assays, are reported in Table 3.

**Table 3:** Detection Limits via LC-MS Analyses of DBS, DSS, VTS, Plasma, Serum, And Urine Assays

					LOD in plasma, serum, or urine (from other studies)			LOD in urine: conventional pretreatment	
Full name	Abbreviation	LOD in DSS, ng/mL (Osteresch et al.^46^)	LOD in DBS, ng/mL (Osteresch et al.^46^)	LOD in VTS, ng/mL (Vidal et al.^30^)	LOD, ng/mL (ng/g)	ref	note	LOD, ng/mL	ref
2’R-Ochratoxin A	2’R-OTA	0.012	0.014	-	0.10	Jaus et al.^100^	Serum	-	-
10-Hydroxyochratoxin A	10-OH-OTA	0.015	0.013	-	-	-	-	-	-
Aflatoxin B_1_	AFB_1_	0.012	0.006	0.040	0.04	Arce-López et al.^101^	Plasma	0.83	Ediagea et al.^102^
Aflatoxin B_2_	AFB_2_	0.013	0.013	0.130	0.07	Arce-López et al.^101^	Plasma	-	-
Aflatoxin G_1_	AFG_1_	0.021	0.014	0.120	0.07	Arce-López et al.^101^	Plasma	-	-
Aflatoxin G_2_	AFG_2_	0.037	0.027	0.150	0.35	Arce-López et al.^101^	Plasma	-	-
Aflatoxin M_1_	AFM_1_	0.017	0.014	0.130	0.18	Arce-López et al.^101^	Plasma	0.06	Solfrizzo et al.^103^
Altenuene	ALT	0.147	0.081	-	-	-	-	0.20	Fan et al.^104^
Alternariol monomethyl ether	AME	0.146	0.146	1.860	-	-	-	0.02	Fan et al.^104^
Alternariol	AOH	0.142	0.142	1.370	-	-	-	0.04	Fan et al.^104^
Beauvericin	BEA	0.014	0.013	-	0.02	Serrano et al.^105^	Plasma	-	-
Citrinin	CIT	0.066	0.051	-	0.02	Jaus et al.^100^	Serum	2.88	Ediagea et al.^13^
Dihydrocitrinone	DH–CIT	0.268	0.270	-	0.10	Jaus et al.^100^	Serum	-	-
Deoxynivalenol	DON	0.263	0.292	0.390	1.94	Arce-López et al.^101^	Plasma	0.80	Solfrizzo et al.^15^
DON-3-glucuronide	DON-3-GlcA	1.287	1.335	0.850	-	-	-	2.25	Ediagea et al.^13^
Enniatin A	EnA	0.002	0.002	-	0.04	Serrano et al.^17^	Plasma	-	-
Enniatin A_1_	EnA_1_	0.006	0.003	-	0.01	Serrano et al.^17^	Plasma	-	-
Enniatin B	EnB	0.001	0.001	-	0.01	Serrano et al.^17^	Plasma	-	-
Enniatin B_1_	EnB_1_	0.004	0.004	-	0.02	Serrano et al.^17^	Plasma	-	-
Fumonisin B_1_	FB_1_	0.521	0.627	1.540	0.20	Arce-López et al.^101^	Plasma	0.05	Solfrizzo et al.^15^
HT-2 toxin	HT-2	1.344	1.396	0.740	2.70	Arce-López et al.^101^	Plasma	0.42	Ediagea et al.^13^
HT-2-toxin-4-glucuronide	HT-2–4-GlcA	0.709	0.713	-	-	-	-	-	-
Ochratoxin A	OTA	0.012	0.014	0.180	0.40	Arce-López et al.^101^	Plasma	0.03	Solfrizzo et al.^15^
Ochratoxin α	OTα	0.014	0.014	0.140	0.10	Jaus et al.^100^	Serum	-	-
T-2 toxin	T-2	0.227	0.205	0.580	0.20	Arce-López et al.^101^	Plasma	0.05	Ediagea et al.^13^
Zearalanone	ZAN	0.273	0.277	-	0.20	Slobodchikova & Vuckovic^69^	Plasma	-	-
Zearalenone	ZEN	0.294	0.289	2.150	1.80	Arce-López et al.^101^	Plasma	1.24	Ediagea et al.^13^

This study also performed stability testing for all 27 mycotoxins and metabolites across a variety of storage conditions (room temperature, 4 °C [refrigerated], and −18 °C [frozen]) and at several time points (1, 5, and 10 weeks for room temperature; 24 weeks for refrigerated and frozen conditions). The aflatoxins were stable at 1 week when stored at room temperature (recovery rates >84%, with most >93%).^46^ However, at 5- and 10 weeks, recovery rates for the aflatoxins decreased to as low as 44% and 17% when stored at room temperature, respectively.^46^ The time-dependent degradation was almost entirely mitigated at 24 weeks when samples were refrigerated (recovery rates >79%, with most >85%) or frozen (recovery rates >94%).^46^

Renaud et al. (2022) produced reference materials of AFB_1_-lysine HSA adducts by spotting known blood volumes (20 μL) onto DBS cards at varying concentrations.^47^ In this study, DBS reference materials were compared to VTS and serum samples. VTS had a high agreement with known serum sample values, while DBS was found to have significant matrix effects (signal suppression). This may have resulted from not using an initial extraction step resulting in protein digestion directly on the DBS cards.^47^ Additionally, VTS with known concentrations ranging from 4 to 50 ng/mL AFB_1_-lysine were highly correlated (*r*^2^ = 0.99) with DBS concentrations (Table 2).^47^ Srinivasan et al. (2022) explored the concordance of AFB_1_-lysine adducts in matching venous and capillary blood samples (not DBS) among 36 study participants.^57^ In this study, the albumin-normalized AFB_1_-lysine adduct concentrations between venous and capillary samples were found to have reasonable agreement (*r* = 0.71).^57^

## Ochratoxins

### Background

OTA is the most toxic of the ochratoxins and can be found in coffee beans, cereals (oats, maize, wheat, and barley), and other food sources.^20,51^ In animal models, OTA has been demonstrated to be nephrotoxic, hepatotoxic, genotoxic, and teratogenic.^20,51^ OTA is classified as a group 2B substance of possible human carcinogenicity by the IARC.^58^ Blood OTA levels are primarily bound to human serum albumin.^20^

A recent HBM study conducted in Spain quantified the plasma levels of 19 mycotoxin biomarkers among 438 individuals and reported OTA to be the most prevalent exposure biomarker.^59^ The LOD of this assay, which used plasma as a sampling matrix, was 0.52 ng/mL and OTA levels were detected in 97.3% of samples (concentration range: undetectable to 45.7 ng/mL, mean: 2.99 ng/mL).^59^ Ochratoxin B was also detectable in 10% of the samples. OTA exposure levels were higher than previous studies which quantified OTA levels in blood or plasma in other regions of Spain (range of means: 0.63–1.19 ng/mL).^59^ Other countries for which OTA levels have been recently reported include Sweden, China, the Czech Republic, Italy, Germany, Portugal, Bangladesh, and Egypt (ranges of mean concentrations: undetectable to 1.21 ng/mL).^59^

### Methods

Cramer et al. (2015) measured OTA in human DBS samples using HPLC-MS/MS.^34^ This study also measured the corresponding thermal degradation product, 2’R-ochratoxin A (2’R-OTA), which can form during the processing of contaminated food, including during coffee roasting.^34^ In this study, venous blood was drawn from 34 coffee drinkers and 16 noncoffee drinkers and 100 μL aliquots were applied to DBS samples.^34^ OTA was detected in 100% of samples from a concentration range of 0.071–0.383 ng/mL (mean: 0.21 ng/mL).^34^ 2’R-OTA was detected in 100% of samples in coffee drinkers with concentrations ranging from 0.021–0.414 ng/mL (mean: 0.11 ng/mL) and was detected in 0% of samples from noncoffee drinkers.^34^ These average concentrations were comparable to previous levels reported in the German adult population (∼0.2 ng/mL).^34^ This assay was developed for 40 samples per batch and analytes were stable for up to 4 weeks when stored at 4 °C.^34^

Osteresch et al. (2016) improved upon this method by investigating the influence of hematocrit, blood spot volume, and DBS venous versus finger-prick blood samples.^48^ OTA and 2’R-OTA levels were analyzed in 8.8 mm punches (∼18.7 μL of blood) using HPLC-MS/MS and compared to values derived from whole DBS spots (∼100 μL of blood).^48^ Spiked DBS samples with known concentrations ranging from 0.05 to 1.00 ng/mL OTA and 2’R-OTA in whole spots were highly correlated (*r*^2^ > 0.99) with concentrations in the 8.8 mm punches.^48^ In addition, 8.8 mm punches were taken from the center of DBS cards, which were spotted with venous blood volumes of 75, 100, and 125 μL.^48^ The blood spot volumes did not influence OTA measurements when the same DBS punch sizes were used, which suggests homogeneous dispersion. Matching venous and capillary DBS measurements were also compared using the same punch sizes, with excellent agreement between matching finger-prick (capillary) and venous DBS values (*r*^2^ = 0.93 and 0.91 for OTA and 2’R-OTA, respectively), suggesting capillary blood is suitable for DBS analyses.^48^

Overall, the assay was sensitive and precise. The limit of detection (LOD) and limit of quantification (LOQ) for both OTA and 2’R-OTA in matrix-free solution were 0.005 ng/mL and 0.013 ng/mL.^48^ The LOD and LOQ in the sampling matrix was only determined for 2’R-OTA and was 0.006 ng/mL and 0.021 ng/mL for the 100 μL whole spot DBS samples, respectively.^48^ For punched DBS samples, the LOD and LOQ for OTA were reported to be 0.008 ng/mL and 0.026 ng/mL, respectively.^48^ Recovery rates were ∼100% for both OTA and 2’R-OTA.^48^ Hematocrit effects were negligible.^48^ This assay was applied to a small German cohort (50 blood samples collected from volunteers during the previous study)^34^ to evaluate the accuracy of the DBS-punching method. OTA and 2’R-OTA levels were determined from both 100 μL whole DBS spots and 8.8 mm punched discs.^48^ OTA had a detection frequency of 100% while 2’R-OTA was detected in 68% (34/50) of the samples.^48^ OTA concentrations derived from whole spots (100 μL blood) and 8.8 mm discs showed strong agreement (*r*^2^ = 0.68 and 0.70 for OTA and 2’R-OTA, respectively).^48^ OTA and 2’R-OTA concentrations in the sample ranged from ∼0.1 to 0.4 ng/mL.^48^ Given the low analyte concentrations investigated, the results suggest that the punching method with smaller blood volumes (∼18.7 μL of blood) is adequate for OTA quantification.^48^

Osteresch et al. (2017) expanded the assay to include several ochratoxins (OTA, 2’R-OTA, Ochratoxin α (OTα), and 10-hydroxyochratoxin A (10-OH-OTA).^46^ Average recovery rates in spiked DBS/DSS samples were close to 100% for all target ochratoxins.^46^ The LOD and LOQ for OTA and 2’R-OTA was higher than reported previously at 0.014 and 0.05 ng/mL, respectively.^46^ However, this assay produced LOQs, which are comparable to LOQs (∼0.03 ng/mL) published in previous methods to detect and quantify OTA.^46,60^ For an additional comparison, a recent assay developed using plasma (not DSS) had a LOD of 0.52 ng/mL and was able to detect OTA in 97.3% of samples.^59^Table 3 contains additional comparisons between the DBS/DSS and other detection methods, including VTS. Both OTA and 2’R-OTA showed only minor matrix effects.^46^

Stability testing revealed that OTA and 2’R-OTA were highly stable when stored at room temperature at 1 week (recovery rate: 99%), 5 weeks (93%), and 10 weeks (89%).^46^ DBS samples that were refrigerated or frozen also had high OTA and 2’R-OTA recovery rates (>90%) at 24 weeks.^46^ In contrast, OTα and 10-OH-OTA showed significant time-dependent degradation when stored at room temperature, with recovery rates as low as 35% for OTα at 10 weeks and 69% for 10-OH-OTA.^46^ Refrigerating the samples mitigated these effects with recovery rates >81% for both analytes at 24 weeks, while freezing nearly eliminated any time-dependent degradation.^46^

This novel multimycotoxin assay was applied in two separate studies reanalyzing previously collected samples.^43,46^ The first study^46^ applied the multimycotoxin assay to 50 DBS samples from a German cohort analyzed previously.^34,48^ Enniatin B (EnB), OTA, and 2’R-OTA were the only detectable mycotoxins and metabolites.^46^ This analysis largely confirmed the positive findings of OTA and 2’R-OTA in DBS samples obtained from the same participants analyzed previously. However, since this method^46^ was found to have lower sensitivity and precision compared to the previously developed methods specific for OTA and 2’R-OTA, some previously quantified samples fell between the assay’s LOD and LOQ.^48^ Comparing OTA and 2’R-OTA concentrations measured previously^48^ to the concentrations measured with the multimycotoxin assay on matching DBS samples showed high agreement,^46^ indicating both methods are reliable.

The second study applied the multimycotoxin assay to 42 DSS samples from workers at a waste management plant in Portugal who were expected to have coexposure to multiple mycotoxins.^43^ DSS samples were created by spotting filter paper with 100 μL of serum. The entire spot was used for analyses using HPLC-MS/MS. A previous analysis of the same serum samples detected aflatoxins with enzyme-linked immunosorbent assays (ELISA) methods,^61^ which had high agreement with approaches using HPLC-FDS for measuring aflatoxin albumin adducts.^55^ This study detected EnB, OTA, and 2’R-OTA at detection frequencies of 100%, 100%, and 82%, respectively (with median concentrations of 0.0481, 0.756, and 0.323 ng/mL, respectively).^43^ These were the same mycotoxins detected in the previous application study involving the German cohort.^46^ The authors speculated that coexposure to multiple mycotoxins likely occurred via different routes of exposure (i.e., occupational exposures for aflatoxins and dietary exposure for the other mycotoxins).^43^ However, since a control group was not used for this study, it was not possible to conclude that EnB, OTA, and 2’R-OTA levels were from dietary exposures.

Vidal et al. developed and validated a VTS-based method for multiple mycotoxins including OTA, AFB_1_, and FB_1_ using ultraperformance liquid chromatography–tandem mass spectrometry (UPLC-MS/MS).^30^ VTSs were prepared by spiking 5 different OTA concentration levels (0.5–12.5 ng/mL) on EDTA-anticoagulated blood samples obtained from a commercial vendor, Rode Kruis Vlaanderen (Ghent, Belgium). Recovery rates were 84–91% depending on spiked concentrations (Table 2). Stabilities were >90% in both the 4 °C and room temperature conditions (Table 2). The VTS assay showed similar accuracy to DBS and liquid blood assays in terms of OTA analyses. Renaud et al. (2022) reported a coefficient of determination between DBS and VTS assays of 0.99.^47^ In addition, Vidal et al. (2022) showed OTA concentrations obtained by the VTS assay were similar to those obtained by whole blood from 20 blood samples. OTA showed good stability when using the VTS assay. After 21 days, stability was close to 100% at room temperature, while it was >90% in 4 °C.^30^ The LOD obtained by the VTS assay^30^ was several times higher than LODs obtained by DBS but lower than the LOD obtained by liquid plasma (Table 3).

## Fumonisins

### Background

Fumonisins (FB) are mycotoxins which can grow in maize and may increase the risk for neural tube defects (NTDs), stunting in children, and carcinogenesis.^3,51^ Exposure to fumonisins may be higher in regions where maize is a staple crop, such as Mexico, Central America,^62^ and South Africa.^3,8^ Fumonisin B_1_ (FB_1_) is likely the most prevalent and toxic to humans, with animal models demonstrating the kidneys and liver to be target organs of effects, including carcinogenicity.^3,51^ A few epidemiologic studies have also found exposure to FB_1_ to be associated with esophageal cancer risk.^3,8^ FB_1_ is classified as a group 2B possible human carcinogen by the IARC.^58^ High-quality data on exposure and health risks are lacking in humans due to difficulty in obtaining reliable and precise exposure assessments.^3^

Riley et al. hypothesized that FB_1_ may exert human health effects (e.g., NTDs) via FB_1_ inhibition of ceramide synthase, which is an important enzyme involved in the synthesis of sphingolipids.^41^ Previous studies in animal models had demonstrated this to be a plausible mechanism to induce NTDs in humans.^62−64^ FB_1_ inhibition of ceramide synthase results in elevated levels of sphinganine 1-phosphate (Sa 1-P) and, to a lesser extent, sphingosine 1-phosphate (So 1-P). Thus, Sa 1-P and the Sa 1-P:So 1-P ratio could be used as biomarkers of effect (i.e., mechanism-based biomarkers) for human exposure to FB_1_. Prior studies had validated the use of urinary FB_1_ (UFB_1_) as an exposure biomarker to fumonisins in the diet.^65^ Prior to these studies, blood-based biomarkers had not been used to measure exposure to fumonisins or to demonstrate evidence of ceramide synthesis inhibition in humans.^41^

### Methods

In a series of experiments, Riley et al. (2015a) developed a DBS assay for estimating FB_1_ exposure by measuring Sa 1-P and So 1-P in DBS samples using HPLC-MS/MS.^41^ The DBS assay was first developed and validated in a mouse model. In mice, oral administration of FB_1_, including at levels sufficient to induce NTDs, was positively related to blood Sa 1-P, So 1-P, and the Sa 1-P:So 1-P ratio (measured in DBS) in a dose-dependent manner.^41^ Sa 1-P and the Sa 1-P:So 1-P ratio also had a dose-dependent relationship with UFB_1_.^41^ In mice, Sa 1-P levels measured in DBS were positively related to Sa levels measured in target tissues, including the liver and kidneys.^41^ Elevated Sa and the Sa:So ratio were also measured in embryonic tissue in mice given oral administration of FB_1_,^41^ thus supporting the mechanism by which FB_1_ might induce NTDs *in vivo*.

After the methods were developed and validated in mice, the assay was applied to a small sample of healthy human volunteers (*n* = 10) from the United States. Volunteers ate maize-based food for three consecutive days (estimated FB_1_ intake: ∼2.94 μg/kg body weight per day). No significant changes (*P* < 0.05) of Sa 1-P and So 1-P levels were detectable in DBS samples collected from these volunteers.^41^ Moreover, there was no significant correlation between UFB_1_ and Sa 1-P:So 1-P ratio across the three sampling time points (n = 25 total samples).^41^ Additional blood samples were obtained from volunteers to conduct smaller laboratory-based experiments in which known blood volumes were spotted onto DBS cards.^41^ From these experiments, it was determined that the LOQ for Sa 1-P and So 1-P was 0.8 pmol using 8 mm DBS punches (∼15–17 μL of blood).^41^ Sa 1-P and So 1-P levels were detectable in spotted human blood volume extracts as low as 2.5 μL.^41^ A model was developed and validated for normalizing Sa 1-P and So 1-P in DBS samples by estimated blood volumes.^41^ Spotting different volumes of blood onto DBS cards had no significant effects on Sa 1-P:So 1-P ratio. Stability testing showed no significant time-dependent degradation for the Sa 1-P and So 1-P biomarkers for 170 days when stored at −20 °C in spotted human DBS samples.^41^

This DBS assay was then applied in a pilot study (2010–2011) conducted in Guatemala to assess the feasibility of using DBS sampling in the field.^41^ In the pilot study, DBS samples were collected from a total of 176 participants (*n* = 76 women and 100 men) from two departments (Chimaltenango and Escuintla) in Guatemala. Chimaltenango and Escuintla were originally selected to compare samples from the expected low and high FB exposure populations, respectively. In other work, maize grown in hotter and dryer climates were determined to have higher FB contamination compared to maize grown at high elevations and colder climates.^66^ However, in this study, both Chimaltenango and Escuintla were ultimately determined to have low levels of FB exposure after urine and maize sampling (estimated total FB intake: ∼0.41 μg/kg of body weight per day). Subsequent analyses of DBS and urinary samples showed no relationship between UFB_1_, Sa 1-P, So 1-P, or the Sa 1-p:So 1-P ratio in both men and women.^41^ The null findings of the pilot field study in Guatemala, as well as the small study involving volunteers from the United States, can likely be attributed to the low FB exposure levels of study participants. The study in the United States was also limited by a very small sample size (*n* = 10 volunteers).^41^ Therefore, detectable concentrations of Sa 1-P and So 1-P in DBS samples may only be seen among populations with chronically elevated FB exposures,^41^ especially if there is a threshold level above which FB inhibits ceramide synthase.

In a follow-up larger field study (2011–2012), urinary and DBS samples were collected from a total of 1,240 women from two low-exposure departments (Chimaltenango, *n* = 439; Escuintla, *n* = 402) and one high-exposure department (Jutiapa, *n* = 399) in Guatemala.^42^ DBS samples were collected from 1,233 of the study participants and used for the analyses of Sa 1-P and So 1-P levels. Urine samples were collected at the same time points and later quantified for FB_1_ (UFB_1_). Urine and DBS samples were collected every 3 months over a one year time frame (4 total sampling time points). Only women were recruited for this study, because future work will investigate whether FB exposure increases the risk for NTDs. Maize in Jutiapa was determined to have total FB and FB_1_ contamination levels that were approximately 5–6 times higher than contamination levels in Chimaltenango and Escuintla.^66^ UFB_1_ had a dose-dependent relationship with estimated dietary FB intake.^66^ The average UFB_1_ levels were in Jutiapa were 2.27 ng/mL compared to 0.38 and 0.26 ng/mL in Chimaltenango and Escuintla, respectively.^66^ Moreover, 75% of the study participants from the high-exposure department (Jutiapa) were estimated to have total FB intake exceeding 2 μg/kg body weight per day, which is considered the provisional maximum tolerable daily intake (PMTDI).^66^ These results confirm that FB exposure was higher in Jutiapa compared to Chimaltenango and Escuintla and that these were exposure levels of possible concern.

Corroborating these findings, Sa 1-P concentrations, and Sa 1-P:So 1-P ratios measured in DBS samples were found to be significantly higher in Jutiapa compared to Chimaltenango or Escuintla.^42^ Individually matched DBS values of Sa 1-P and Sa 1-P:So 1-P ratios were positively associated with UFB_1_ measurements,^42^ which provides further validation of the exposure biomarker. Moreover, these results were demonstrated across sampling time points. The data reported also support the hypothesis of a threshold effect by which Sa 1-P concentrations (>0.88 nmol/mL) and Sa 1-P:So 1-P ratios (>0.36) are significantly associated with elevated UFB_1_ concentrations above these thresholds.^42^

A confirmatory field study (February–March 2013) was conducted in one low exposure department (Sacatepéquez, *n* = 100) and two high exposure departments (Santa Rosa, *n* = 100, and Chiquimula, *n* = 99).^42^ A total of 299 study participants (all women) were included. Maize contamination levels were confirmed to be significantly higher in Santa Rosa and Chiquimula compared to Sacatepéquez. For example, using maize sampling, the estimated average FB_1_ intake exceeded the PMTDI in 68% and 85% of study participants from Santa Rosa and Chiquimula, respectively.^42^ Similar FB exposure estimates were concluded from UFB_1_ measurements. Corroborating the prior field study, a dose-dependent relationship between total FB intake and urinary FB_1_ was demonstrated.^42^ Analyzing the DBS samples (*n* = 299) showed significantly higher concentrations of Sa 1-P and ratios of Sa 1-P:So 1-P in Chiquimula and Santa Rosa compared to Sacatepéquez.^42^ As in the prior study, individually matched Sa 1-P concentrations and Sa 1-P and Sa 1-P:So 1-P ratios were significantly correlated with UFB_1_ levels.^42^ Moreover, the results largely confirmed the threshold effect reported in the previous (2011–2012) field study.^42^

The results from these three Guatemalan field studies (2010–2011 pilot study; 2011–2012; and 2012–2013) suggest that individuals with UFB_1_ levels >0.1 ng/mL have an increased risk for exceeding the PMTDI FB exposure level, while individuals >0.5 ng/mL will almost certainly exceed the PMTDI.^42^ Moreover, these studies support the hypothesis that FB_1_ inhibits the ceramide synthesis in humans. The next step will be to investigate associations among UFB_1_, Sa 1-P, and Sa 1-P:So 1-P ratios and incident NTD cases in a prospective cohort study. The studies by Riley et al.^41,42,66^ demonstrated that DBS sampling is feasible for large-scale field studies measuring exposure to fumonisins (with simultaneous collection of urine samples). It is important to note, however, that FB was not directly measured in the DBS samples in these studies.

The multimycotoxin assay discussed previously^46^ included FB_1_, raising the possibility of directly measuring FB_1_ in DBS samples in future field studies rather than measuring UFB_1_ in urine samples. The reported LOD and LOQ of the DBS assay for FB_1_ was 0.627 ng/mL and 2.5 ng/mL, respectively.^46^ The average recovery rate was 97% in spiked DBS samples and 61% in DSS samples.^46^ FB_1_ was stable in DBS samples when stored at −18 °C for 24 weeks. However, significant degradation was apparent when stored at 20 °C for 5 (55% recovery rate) and 10 weeks (37% recovery rate).^46^ Using these methods, FB_1_ was not detectable in DBS samples from the small German cohort discussed previously (*n* = 50).^46^ The inability to detect FB in this sample would be expected, since FB exposure was likely low in this population. Similar findings were reported by Riley et al. in their initial studies among only low-exposure groups,^41^ and could reflect inadequate sensitivity of the assay. Applying this assay to DBS samples collected to samples from a population with likely higher levels of exposure to FB, as was done in Riley et al. (2015b),^42^ should be an objective for future studies.

Vidal et al. spiked samples in the VTS assay with 5 different FB1 concentrations (10–250 ng/mL).^30^ Compared with the multimycotoxin DBS assay,^46^ this VTS assay had lower sensitivity and stability. The LODs obtained by the VTS assay were several times higher than the LODs obtained by DBS or urine assays (Table 3). While higher concentrations of spiked FB_1_ (50–250 ng/mL) were relatively stable (i.e., >90%), recovery rates of FB_1_ at lower concentrations (10 ng/mL) were <70% at 21 weeks when refrigerated at 4 °C.

## Discussion

This review summarizes recent advances in measuring mycotoxins in human DBS/DSS samples and VTS. The studies reviewed have focused on method development with applications in relatively small populations, which have not yet been applied to larger-scale population-based studies. While Riley et al. demonstrated feasibility of performing DBS sampling for measuring mycotoxin exposure biomarkers in larger-scale field studies,^42^ studies which have applied the multimycotoxin DBS/DSS assay by Osteresch et al. (2017) created DSS samples from venous blood.^43−45^ The multimycotoxin assay adapted for DBS/DSS^46^ represents a powerful method to detect an array of important mycotoxin exposure biomarkers and can be useful for applications in large-scale temporal biomonitoring and surveillance studies. Although the assay’s sensitivity and precision were slightly lower for OTA, the assay performed well overall with recoveries greater than 90% for most target analytes.^46^ While other multimycotoxin assays have been developed using human blood, plasma, and urine,^67−69^ this was the first assay adapted for DBS/DSS analyses and had high recovery rates.^10^ However, the assay was applied to a population with undetectable exposures for most biomarkers (except for OTA, 2’R-OTA, and Enniatin B) and should be validated in populations with wider ranges of mycotoxin exposures in future studies. In addition, this sample^46^ may be underpowered and larger sample sizes would likely result in higher detection frequencies for many of the mycotoxin biomarkers analyzed.

Multimycotoxin assays^30,46^ should be expanded to include additional mycotoxins of interest, including trichothecenes, patulin (PAT), and citrinin (CIT), which are important mycotoxins found in many legislative efforts to reduce human exposures.^7^ In addition, mycotoxins such as tenuazonic acid (TeA), produced by the *Alternaria* species, have been measured in DBS samples using pig’s whole blood with low levels of detection.^70^ ZEA are also important mycotoxins included in the multimycotoxin assays developed by Osteresch et al.^46^ and Vidal et al.^30^ which can result in adverse reproductive effects in animals, including infertility, embryo death, and testosterone attrition.^71−73^

In general, blood-based biomarkers have the potential to better capture long-term mycotoxin exposure, while mycotoxin exposure biomarkers measured in the urine are more representative of acute exposures.^10^ This is especially true for blood protein adduct biomarkers (e.g., hemoglobin or albumin adducts), which reflect an individual’s exposure history integrated over weeks to months.^12^ Currently, urine is a preferred sampling matrix in field studies for many analytes due to its noninvasive nature and acceptability among study participants.^7^ However, DBS sampling represents a field-friendly complement to urine sample collection with a high level of acceptability by study participants,^74−76^ especially in settings where DBS sampling has been used for other purposes, such as for the monitoring of HIV antiretroviral treatment.^77^ Moreover, the use of minimally invasive sampling methods may facilitate repeated sampling from the same individual in longitudinal cohort studies, which allows for determining ICCs to inform reliability of mycotoxin exposure biomarkers.^40^

Since many mycotoxin exposure biomarkers are better detected in blood (e.g., OTA, 2’R-OTA, EnB^46^) and others are better detected in urine (e.g., deoxynivalenol-3-glucoronide^46^ and possibly fumonisins^66^), the field collection of both biological specimens is optimal when feasible. Table 3 shows that alternariol monomethyl ether (AME), alternariol (AOH), and FB_1_ have high detection capacity in urine (i.e., LOD < 0.1 ng/mL) while AFB_1_, AFB_2_, AFG_1_, and BEA have high detection capacity in plasma (i.e., LOD < 0.1 ng/mL). A multimycotoxin assay has also recently been developed for dried urine spots.^78^ Furthermore, Schmidt et al. (2021) incorporated online solid-phase extraction with HPLC-MS/MS for detecting mycotoxins biomarkers to achieve greater analytical sensitivity compared to other extraction methods (Table S1 in the Supporting Information).^79^ With further validation and adoption of DBS sampling for measuring chronic human exposure to mycotoxins, additional research questions may be pursued, such as potential additive or synergistic effects between mycotoxin coexposures,^8,43^ other environmental exposures (e.g., trace elements, organic pollutants, and endocrine disrupting chemicals),^25^ and associations with systems biology (e.g., using omics).^10,80^ In addition, animal studies have suggested that aflatoxin and fumonisins coexposures can synergistically increase the risk for developing hepatocellular carcinoma.^4^ Because coexposure to these toxins are common in many countries in Africa^8^ and Central America, multimycotoxin assays adapted to DBS would have significant epidemiologic utility.

Global awareness among key stakeholders is growing for strengthening food safety measures by detecting and reducing mycotoxin contamination levels.^4,81^ Methods that quantify contamination levels in the food supply (i.e., upstream sources) should be coupled with high-throughput, ultrasensitive, and reliable public health tools that quantify levels of human exposures (i.e., downstream effects of contamination in the food supply). DBS sampling can extend surveillance to rural subsistence farming communities, which are at greatest risk of exposure.^8^ Other advantages of using biological sampling compared to food sampling alone include the heterogeneous distribution of mycotoxins in foodstuffs, as well as the ability to capture additional routes of exposure (e.g., inhalation and intradermal) and contamination that might arise from methods of food preparation.^43^ Recent innovations in detecting mycotoxin contaminants in food matrices include aptasensors, which present a low-cost, high-throughput, ultrasensitive approach for improving surveillance across the food supply chain.^82^ A novel, complementary approach uses dried extract spots (DES), which allows for sample collection of food matrices by minimally trained personnel and centralized laboratory processing for precise quantification of contaminants.^83^ Future work may also seek to evaluate the interchangeability of capillary and venous mycotoxin biomarkers to develop point of care (POC) devices, as was demonstrated in a small sample size (36 reproductive age women in Uganda) for albumin-normalized AFB_1_-lysine adducts.^57^

VTS presents another minimally invasive sampling technique that may have utility. In particular, concentrations of AFB1 or OTA collected from VTS had higher reproducibility^84^ than those collected from DBS (Table 2).^35,48^ VTS also allows for easier laboratory blood extraction compared to standard DBS methods.^47^ The use of VTS facilitates sample identification during extraction, but this is not the case with DBS since it cannot be identified once it has been punched.^85,86^ Vidal et al. (2021) validated a multimycotoxin VTS ultraperformance liquid chromatography- tandem mass spectrometry (UPLC-MS/MS) assay for 24 mycotoxins, including aflatoxins, ZEA, ochratoxins, and fumonisins (Table 3).^30^ For multimycotoxins assays, VTS requires 0.25 mL of extraction solution for sonication,^30^ whereas DBS requires 2 mL^46^ (Section S1 of the Supporting Information). VTS values were found to be stable for at least 21 days with refrigeration or at room temperature.^30^ VTS-based procedures had high recovery rates and stability (Table 2), while LODs of the VTS assay were approximately one order higher than those of the DBS- or liquid plasma-based procedures for ochratoxins, aflatoxins, and fumonisins (Table 3). These method validation results suggest that VTS may serve as an alternative to DBS or conventional venous sampling to perform quantitative screening of the exposure of these mycotoxins for highly exposed populations. However, future work is needed to improve the detection limits of the VTS assays.

At present, the most significant disadvantage of VTS is its relatively high cost compared to that of DBS sampling, therefore hindering its ability to expand HBM and environmental epidemiologic studies at scale. VTM is currently offered as two commercially available devices: the Mitra device by Neoteryx (which uses patented volumetric absorptive microsampling technology; VAMS) and the TASSO-M20 device by Tasso, Inc.^87^ In addition, VTS is limited to commercially available sample volumes (e.g., ∼10 μL), which may not be sufficient for certain biomarkers such as aflatoxin adducts to human serum albumin.^47^ Many DBS assays for environmental exposure biomarkers (e.g., cotinine) were found to have minimal hematocrit effects.^25,88^ Among mycotoxin DBS assays, hematocrit effects were negligible for analyses of OTA.^48^ Therefore, VTS may present the greatest value for therapeutic drug monitoring and pharmacokinetic studies where hematocrit effects are most significant.^85,89^ Future work validating DBS assays should continue to investigate hematocrit effects and elucidate the value of VTS versus DBS/DSS sampling on a biomarker-by-biomarker basis.

Overall, the development of field-friendly sampling methods^87,90^ will facilitate longitudinal cohort studies investigating mycotoxins and associated health risks, for which there are currently very few studies.^13^ Due to its low cost and convenience, DBS sampling is well-suited for extending temporal biomonitoring and surveillance studies in low-resource settings, where mycotoxin exposures and associated health risks are the most prevalent.^10^ Scaling up minimally invasive sampling methods for the monitoring of population-level exposures to mycotoxins can elucidate global health inequities^91,92^ and direct efforts for reducing population exposure levels. In regions where exposure to mycotoxins is endemic, exposure is lifelong and may start *in utero.*(3) In high-resource countries, low socioeconomic status may also present increased risk for exposure to mycotoxins; for example, among occupants of water-damaged buildings.^93,94^ Importantly, multimycotoxin assays adapted to DBS/DSS analyses represent a unique and invaluable opportunity to advance studies elucidating the human “exposome”,^10,95^ which represents the totality of human exposures from conception onward.

DBS/DSS multimycotoxin assays can also be applied to epidemiologic studies utilizing archived newborn dried blood spots^25^ and in studies involving infants and young children.^35^ Aflatoxins are lipophilic (i.e., polar) and can exert effects *in utero*. For example, Turner et al. (2007) reported that aflatoxin-albumin adducts were detected from half of the cord blood samples collected from 138 Gambian infants with levels ranging from 5.0–189.6 pg/mg.^96^ In Gambia, aflatoxin-albumin adducts were found in more than 80% of tested infants between 3 and 9 years old, and blood levels of aflatoxin-albumin adducts were measured up to 720 pg/mg.^97−99^

An important limitation of minimally invasive sampling approaches is the analytical challenges associated with small sample volumes, which limit the ability to analyze samples for additional biomarkers. This limitation may be overcome by continued innovations in microsampling methods and continued improvements in analytical sensitivity and precision of mass spectrometry instrumentation^87,90^ as well as multimycotoxin assays.^30,46^ Advancing the science of DBS/DSS sampling and VTS for measuring exposure to mycotoxins and implementing these approaches widely as public health tools should be a research priority to promote global health equity. Additional work is needed to identify appropriate and sustainable financing mechanisms and implementation strategies to incorporate DBS/DSS sampling and VTS into global health surveillance systems for monitoring population-level exposures to mycotoxins in regions where exposures are endemic and in regions where exposures are expected to increase with global climate change.^16,18,54^

## Environmental Implications

Minimally invasive sampling assays have been developed and validated for important mycotoxins including ochratoxins, aflatoxins, and fumonisins. DBS/DSS sampling and VTS can facilitate the collection of longitudinal epidemiologic data on human exposure to mycotoxins and elucidate associated health risks, especially in low-resource settings. Low cost and field-friendly (minimally invasive) approaches are needed to evaluate mycotoxin exposures and direct mitigation efforts. Several of the DBS/DSS and VTS assays described in this review have sufficient validation for deployment in the field (Table S2 in the Supporting Information) and should be scaled-up for surveillance efforts and population health studies. Multimycotoxin assays should be expanded to include other mycotoxins of interest. DBS/DSS and VTS measurements should be further validated against gold-standard venous blood values, and stability concerns across different storage conditions (e.g., high heat and humidity) need to be addressed on a biomarker-by-biomarker basis. Continued improvements in detection limits, reliability, and analyte stability for multimycotoxin assays can facilitate widespread implementation of DBS/DSS and VTS sampling to reduce global inequities in mycotoxin exposures.

## Supporting Information Available

Details on literature search strategy, blood extraction and pretreatment procedures, and Tables S1 and S2 (PDF)
