# Supplementary material for: Advancing Global Health Surveillance of Mycotoxin Exposures using Minimally Invasive Sampling Techniques: A State-of-the-Science Review
Source: Environ Sci Technol. 2024 Feb 14;58(8):3580–94. doi: 10.1021/acs.est.3c04981 (PMC10903514; doi:10.1021/acs.est.3c04981)
Supplement: Supplementary file 1 — es3c04981_si_001.pdf [file es3c04981_si_001.pdf]

Supporting Information for

Advancing Global Health Surveillance of Mycotoxin Exposures using Minimally Invasive Sampling Techniques: A State-of-the-Science Review

Environmental Science and Technology

[1]*Tyler A. Jacobson, BBA, [1]*Yeunook Bae, PhD, [2]Jasdeep S. Kler, MPH, [1]Ramsunder Iyer, PhD, [1]Runze Zhang, MPH, [1]Nathan D. Montgomery, PhD, [3]Denise Nunes MS, RN, MSLIS, [4]Joachim D. Pleil, PhD, and[1†]William E. Funk, PhD (*Contributed equally)

[1]Department of Preventive Medicine, Northwestern University Feinberg School of Medicine, Chicago, IL, USA
[2]University of Michigan Medical School, Ann Arbor, MI, USA
[3]Galter Health Sciences Library, Northwestern University Feinberg School of Medicine, Chicago, IL, USA
[4]Department of Environmental Sciences and Engineering, Gillings School of Public Health, University of North Carolina, Chapel Hill, NC, USA

[†]Corresponding author: William E. Funk, E-mail: w-funk@northwestern.edu, Phone: (312) 503-4092, 680 N Lake Shore Dr., Suite 1400, Chicago, Illinois 60611, United States.

Supporting Information contains 12 pages with 2 Sections and 2 Tables

Contents

Section S1. Details on Literature Search Strategy S3

Section S2. Blood Extraction and Pretreatment Procedures from Dried Blood Spots (DBS), Dried Serum Spots (DSS), or Volumetric Tip Microsampling (VTS). S7

Tables

Table S1. Detection limits through mass spectrometry analyses of DBS and urine assays.. S9

Table S2. Summary of developed DBS method and for each mycotoxin exposure S10

References S11

Section S1. **Details on Literature Search Strategy.**

Inclusion criteria:

- Developed, validated, and/or applied methods to measure mycotoxin exposure biomarkers. We included two reports which measured a biomarker of effect,[1] since this biomarker is specific to environmental exposure to fumonisins.

- Used a minimally invasive sampling technique, including dried blood spot (DBS), dried serum spot (DSS), and volumetric tip microsampling (VTS) / volumetric absorptive microsampling (VAMS) assays.

- Assays were developed for human blood samples (not animal samples).

Our systematic search of the literature conducted in March 2022 was designed to identify all studies measuring environmental exposure biomarkers (including mycotoxins) in dried blood spot (DBS) samples. This search was conducted in PubMed, Embase, and CINAHL and identified 1620 articles after de-duplication. Details of the systematic search have been published in our prior review of environmental exposure biomarkers, which included environmental tobacco smoke, trace elements, and persistent organic pollutants (Jacobson et al., 2022). Specific search terms and results can be found in the Supplemental Information of Jacobson et al., 2022. Title and Abstract screening were performed in duplicate using the screening platform, Rayyan. This original systematic search identified **5 published reports** measuring mycotoxin exposure biomarkers in DBS samples.

- Cramer, B.; Osteresch, B.; Muñoz, K. A.; Hillmann, H.; Sibrowski, W.; Humpf, H. U. Biomonitoring using dried blood spots: detection of ochratoxin A and its degradation product 2'R-ochratoxin A in blood from coffee drinkers. *Molecular Nutrition & Food Research* **2015**, *59*(9), 1837.
- Osteresch, B.; Cramer, B.; Humpf, H. U. Analysis of ochratoxin A in dried blood spots - Correlation between venous and finger-prick blood, the influence of hematocrit and spotted volume. *Journal of Chromatography B* **2016**, *1020*, 158.
- Osteresch, B.; Viegas, S.; Cramer, B.; Humpf, H. U. Multi-mycotoxin analysis using dried blood spots and dried serum spots. *Analytical and Bioanalytical Chemistry* **2017**, *409*(13), 3369.
- Xue, K. S.; Cai, W. J.; Tang, L. L.; Wang, J. S. Aflatoxin B-1-lysine adduct in dried blood spot samples of animals and humans. *Food and Chemical Toxicology* **2016**, *98*, 210.
- Riley, R. T.; Showker, J. L.; Lee, C. M.; Zipperer, C. E.; Mitchell, T. R.; Voss, K. A.; Zitomer, N. C.; Torres, O.; Matute, J.; Gregory, S. G.; Ashley-Koch, A. E.;

Maddox, J. R.; Gardner, N.; Gelineau-Van Waes, J. B. A blood spot method for detecting fumonisin-induced changes in putative sphingolipid biomarkers in LM/Bc mice and humans. *Food Additives & Contaminants: Part A* **2015**, *32*(6), 934.

We adapted and re-ran this search using PubMed in October 2023 to identify all reports of measuring mycotoxins in either DBS, DSS, or VAMS/VTS samples (i.e., minimally invasive sampling methods).

PubMed search (conducted in October 2023 with no date filter):

Concept 1: Minimally invasive sampling methods, including dried blood spot (DBS), dried serum spot (DSS), and Volumetric Tip Microsampling (VTS) / Volumetric Absorptive Microsampling (VAMS).

Dried Blood Spot Testing [Mesh] OR DBS[Title] OR "Guthrie card*" [Title/Abstract] OR "Guthrie paper*" [Title/Abstract] OR "dried blood" [Title/Abstract] OR "dried whole blood spot" [Title/Abstract] OR "blood blot*" [Title/Abstract] OR "Dried whole blood spot*" [Title/Abstract] OR "dried serum spot" [Title/Abstract] OR "blood spot*" [Title/Abstract] OR "filter paper*" [Title/Abstract] OR "Volumetric absorptive microsampling" OR "Minimally invasive sampling" [Title/Abstract] OR "Volumetric tip" [Title/Abstract] OR "Microsampling" [Title/Abstract]

AND

Concept 2: Environmental exposure biomarkers, adapted to capture all mycotoxin exposure biomarkers.

"Biomarkers" [Majr] OR Environmental Biomarkers [Mesh] OR Biomarker* [Title/Abstract] OR "environmental biomarkers" [Title/Abstract] OR "exposure biomarker*" [Title/Abstract] OR Environmental Pollutants [Majr] OR Environmental Exposure [Mesh] OR "biological monitoring" [Title/Abstract] OR "Exposome" [Title/Abstract] OR "biomonitoring" [Title/Abstract] OR "Mycotoxin*" OR "carcinogen" [Title/Abstract] OR "Toxicant" [Title/Abstract] OR "ochratoxin*" [Title/Abstract] OR "aflatoxin*" [Title/Abstract] OR "Altenuene" [Title/Abstract] OR "Beauvericin" [Title/Abstract] OR "Dihydrocitrinone" [Title/Abstract] OR "Deoxynivalenol" [Title/Abstract] OR "DON-3-glucuronide" [Title/Abstract] OR "Enniatin*" [Title/Abstract] OR "Zearalanone" [Title/Abstract]

This search resulted in **1,459 records** in PubMed. We manually performed title and abstract screening in PubMed. We identified **9 reports** related to methods development, validation, or application of minimally invasive sampling methods for mycotoxin exposure biomarkers. We identified two additional reports from searching the reference

lists of included studies and following citation trails of studies that met our inclusion criteria. We used both Web of Science and Google Scholar to follow citation trails for each article that met our inclusion criteria. A total of **11 reports** met our inclusion criteria, as detailed below.

Primarily Methods Development and Validation:

- Cramer, B.; Osteresch, B.; Muñoz, K. A.; Hillmann, H.; Sibrowski, W.; Humpf, H. U. Biomonitoring using dried blood spots: detection of ochratoxin A and its degradation product 2'R-ochratoxin A in blood from coffee drinkers. *Molecular Nutrition & Food Research* **2015**, *59*(9), 1837.
- Osteresch, B.; Cramer, B.; Humpf, H. U. Analysis of ochratoxin A in dried blood spots - Correlation between venous and finger-prick blood, the influence of hematocrit and spotted volume. *Journal of Chromatography B* **2016**, *1020*, 158.
- Osteresch, B.; Viegas, S.; Cramer, B.; Humpf, H. U. Multi-mycotoxin analysis using dried blood spots and dried serum spots. *Analytical and Bioanalytical Chemistry* **2017**, *409*(13), 3369.
- Xue, K. S.; Cai, W. J.; Tang, L. L.; Wang, J. S. Aflatoxin B-1-lysine adduct in dried blood spot samples of animals and humans. *Food and Chemical Toxicology* **2016**, *98*, 210.
- Riley, R. T.; Showker, J. L.; Lee, C. M.; Zipperer, C. E.; Mitchell, T. R.; Voss, K. A.; Zitomer, N. C.; Torres, O.; Matute, J.; Gregory, S. G.; Ashley-Koch, A. E.; Maddox, J. R.; Gardner, N.; Gelineau-Van Waes, J. B. A blood spot method for detecting fumonisin-induced changes in putative sphingolipid biomarkers in LM/Bc mice and humans. *Food Additives & Contaminants: Part A* **2015**, *32*(6), 934.
- Riley, R. T.; Torres, O.; Matute, J.; Gregory, S. G.; Ashley-Koch, A. E.; Showker, J. L.; Mitchell, T.; Voss, K. A.; Maddox, J. R.; Gelineau-van Waes, J. B. Evidence for fumonisin inhibition of ceramide synthase in humans consuming maize-based foods and living in high exposure communities in Guatemala. *Molecular Nutrition & Food Research* **2015**, *59*(11), 2209.
- Renaud, J. B.; Walsh, J. P.; Sumarah, M. W. Optimization of Aflatoxin B1-Lysine Analysis for Public Health Exposure Studies. *Toxins* **2022**, *14*(10), 672.
- Vidal, A.; Belova, L.; Stove, C.; De Boevre, M.; De Saeger, S. Volumetric Absorptive Microsampling as an Alternative Tool for Biomonitoring of Multi-Mycotoxin Exposure in Resource-Limited Areas. *Toxins (Basel)* **2021**, *13*(5), 345.

Primarily Application of Previously Developed and Validated Assays:

- Viegas, S.; Osteresch, B.; Almeida, A.; Cramer, B.; Humpf, H.-U.; Viegas, C. Enniatin B and ochratoxin A in the blood serum of workers from the waste management setting. *Mycotoxin Research* **2018**, *34*(2), 85.
- Warensjö Lemming, E.; Montano Montes, A.; Schmidt, J.; Cramer, B.; Humpf, H.-U.; Moraeus, L.; Olsen, M. Mycotoxins in blood and urine of Swedish adolescents—possible associations to food intake and other background characteristics. *Mycotoxin Research* **2020**, *36*(2), 193.
- Penczynski, K. J.; Cramer, B.; Dietrich, S.; Humpf, H.-U.; Abraham, K.; Weikert, C. Mycotoxins in Serum and 24-h Urine of Vegans and Omnivores from the Risks and Benefits of a Vegan Diet (RBVD) Study. *Molecular Nutrition & Food Research* **2022**, *66*(6), 2100874.

Our search strategy has the following limitations. We searched terms as subject headings or within the title and abstracts. Therefore, if an article discussed terms related to either concept 1 (minimally invasive sampling methods) or concept 2 (exposure biomarkers/mycotoxins) above but did not include these terms in the title or abstract, our search may not have captured these articles. We used Google Scholar and Web of Science to follow citation trails of reports identified from our initial search. For our search strategy to not capture a relevant article, it would have had to not cite any of the previous studies which developed methods for DBS/DSS/VTS mycotoxin analyses (identified by our two systematic searches in March 2022 and October 2023).

Section S2. Blood Extraction and Pretreatment Procedures from Dried Blood Spots (DBS), Dried Serum Spots (DSS), or Volumetric Tip Microsampling (VTS).

Crame et al. (2015)[2] ***& Osteresch et al. (2016)***[3]

- Target Mycotoxins: OTA, 2'R-OTA
- Procedures:
  1. Sonicate (1 hr) with 1-mL extraction solution (water/acetone/ACN = 6/7/7 (v/v/v))
  2. Evaporate (60 °C) under reduced pressure.
  3. Reconstitute with 100 µL solvent (water/MeOH/formic acid = 60/40/0.1 (v/v/v)).
  4. Centrifuge (10 min, 3,000 g)
  5. Transfer 40 µL to LC vial.

Osteresch et al. (2017)[4]

- Target Mycotoxins: *Multi-mycotoxin*
- Procedures:
  1. Sonicate (0.5 hr) with 2-mL extraction solution (water/acetone/ACN = 6/7/7 (v/v/v))
  2. Evaporate (50 °C) under reduced pressure.
  3. Reconstitute solvent (volume note specified) (water/ACN/acetic acid = 95/5/0.1 (v/v/v)).
  4. Centrifuge (10 min, 22,000 g)
  5. Transfer 30 µL to LC vial.

Xue et al. (2016)[5]

- Target Mycotoxins: *AFB_1-lysine*
- Procedures:
  1. Agitate (0.5 hr) with PBS buffer
  2. Digest (37 °C, 3 hr) the part of the eluants (500 µL) with pronase (enzyme/protein=1/4)
  3. Purify by SPE (Waters MAX SPE cartridges):
     I think the authors used '2% formic acid in MeOH' eluent.
  4. Vacuum-dry the 1-mL eluates (2% formic acid in MeOH)
  5. Reconstitute with 150 µL solvent (25% MeOH).
  6. Periodically vortex to prevent solidification.
  7. Transfer to LC vial.

Riley et al. (2015a,b)[1]

- Target Mycotoxins: *Sa-1-P, So-1-P*
- Procedures:
  1. Agitate with 1-mL extraction solution (water/ACN = 1/1 (v/v) w 5% formic acid)
  2. Sonicate (1 hr, 50 °C)

3. Gently shake (3 hr)
4. Filter centrifuge (4,500 rcf, 10 min) the 0.7-mL extractant w nylon centrifuge tube.
    a. Wash three time with 1.5 mL solution (water/ACN = 1/1 (v/v) w 5% formic acid)
5. Dilute with solution (water/ACN = 1/1 (v/v) w 5% formic acid)
6. Transfer to LC vial.

Viegas et al. (2018)[6]

- Target Mycotoxins: *AFB_1*
- Procedures:
    1. Sonicate (0.5 hr) with 1-mL extraction solution (water/acetone/ACN = 6/7/7 (v/v/v))
    2. Evaporate (60 °C) 800-µL aliquot.
    3. Reconstitute with solvent.
    4. Transfer 40 µL to LC vial.

Renaud et al. (2022)[7]

- Target Mycotoxins: *AFB_1-lysine*
- Procedures:
    1. Incubate (50 °C, 4 hr) with 171.5-µL solution (PBS buffer, water, pronase, pH 7.5)
    2. Remove supernatant
    3. Add MeOH (200 uL) to quench the reaction.
    4. Transfer 200 µL to LC vial.

Vidal et al. (2021)[8]

- Target Mycotoxins: *Multi-mycotoxin*
- Procedures:
    1. Sonicate (20 min) with 0.25-mL extraction solution (water/ACN/acetic acid = 59/40/1 (v/v/v))
    2. Agitate (30 min, room temp.)
    3. Evaporate with N_2 gas.
    4. Reconstitute with 50 µL solvent (MeOH/water = 6/4 (v/v)).
    5. Vigorously vortex and centrifuge (10 min, 5,000g)
    6. Transfer 40 µL to LC vial.

Table S1. Detection limits through mass spectrometry analyses of DBS and urine assays.

Abbreviate	LOD in DSS, ng/mL (Osteresch *et al.*[4])	LOD in DBS, ng/mL (Osteresch *et al.*[4])	LOD in Urine, ng/mL: Conventional Pretreatment			LOD in Urine, ng/mL: · online-SPE · Instrument: UHPLC-MS/MS (Schmidt *et al.*[9])
			LOD (ng/mL)	Instrument	Reference	
2'R-OTA	0.012	0.014	-	-	-	-
10-OH-OTA	0.015	0.013	-	-	-	-
AFB_1	0.012	0.006	0.83	LC–MS/MS	Ediagea *et al.*[10]	
AFB_2	0.013	0.013	-	-	-	-
AFG_1	0.021	0.014	-	-	-	-
AFG_2	0.037	0.027	-	-	-	-
AFM_1	0.017	0.014	0.06	LC–MS/MS	Solfrizzo *et al.*[11]	0.007
ALT	0.147	0.081	0.20	UHPLC-MS/MS[a]	Fan *et al.*[12]	0.210
AME	0.146	0.146	0.02	UHPLC-MS/MS	Fan *et al.*[12]	0.020
AOH	0.142	0.142	0.04	UHPLC-MS/MS	Fan *et al.*[12]	0.270
BEA	0.014	0.013	-	-	-	-
CIT	0.066	0.051	2.88	LC–MS/MS	Ediagea *et al.*[10]	-
DH-CIT	0.268	0.270				0.070
DON	0.263	0.292	0.80	LC–MS/MS	Solfrizzo *et al.*[11]	-
DON-3-GlcA	1.287	1.335	2.25	LC–MS/MS	Ediagea *et al.*[10]	-
EnA	0.002	0.002	-	-	-	-
EnA_1	0.006	0.003	-	-	-	-
EnB	0.001	0.001	-	-	-	-
EnB_1	0.004	0.004	-	-	-	-
FB_1	0.521	0.627	0.05	LC–MS/MS	Solfrizzo *et al.*[11]	0.004
HT-2	1.344	1.396	0.42	LC–MS/MS	Ediagea *et al.*[10]	-
HT-2-4-GlcA	0.709	0.713	-	-	-	-
OTA	0.012	0.014	0.03	LC–MS/MS	Solfrizzo *et al.*[11]	0.004
OTα	0.014	0.014	-	-	-	-
T-2	0.227	0.205	0.05	LC–MS/MS	Ediagea *et al.*[10]	-
ZAN	0.273	0.277	-	-	-	-
ZEN	0.294	0.289	1.24	LC–MS/MS	Ediagea *et al.*[10]	0.010

[a] Ultrahigh-performance-liquid chromatography tandem mass spectrometry

Table S2. Summary of developed DBS method and for each mycotoxin exposure. The unit 'mL as blood or DBS' were converted into 'mL as plasma'. Here the hematocrit was regarded as 0.45. The unit 'ng/g as albumin' were converted into 'ng/mL as plasma'. The albumin density in plasma was assumed as 0.073 g/mL.

Exposure (Biomarker)	DBS Method		Applied	
	LOQ [a]	Reference	Concentration	Reference
OTA	For 100-µL DBS: 0.038 ng/mL as plasma	Cramer *et al.*[2]	97.5% of cohorts were >1.46 ng/mL as plasma	Leroy *et al.*[13]
AFB_1-lysine	0.029 ng/mL as plasma	Xue *et al.*[5]	97.5% of cohorts were >1.46 ng/mL as plasma	Leroy *et al.*[13]
Sa-1-P	0.8 pmol in 8-mm spot = 32.6 ng/ml as plasma	Riley *et al.*[1]	97.5% of cohorts were >41.8 ng/mL as plasma	Becker *et al.*[14]

[a]LOQs were converted from reported LODs by multiplying by 3.33.

References

1. Riley, R. T.; Showker, J. L.; Lee, C. M.; Zipperer, C. E.; Mitchell, T. R.; Voss, K. A.; Zitomer, N. C.; Torres, O.; Matute, J.; Gregory, S. G.; Ashley-Koch, A. E.; Maddox, J. R.; Gardner, N.; Gelineau-Van Waes, J. B. A blood spot method for detecting fumonisin-induced changes in putative sphingolipid biomarkers in LM/Bc mice and humans. *Food Additives & Contaminants: Part A* **2015**, *32*(6), 934.

2. Cramer, B.; Osteresch, B.; Muñoz, K. A.; Hillmann, H.; Sibrowski, W.; Humpf, H. U. Biomonitoring using dried blood spots: detection of ochratoxin A and its degradation product 2'R-ochratoxin A in blood from coffee drinkers. *Molecular Nutrition & Food Research* **2015**, *59*(9), 1837.

3. Osteresch, B.; Cramer, B.; Humpf, H. U. Analysis of ochratoxin A in dried blood spots - Correlation between venous and finger-prick blood, the influence of hematocrit and spotted volume. *Journal of Chromatography B* **2016**, *1020*, 158.

4. Osteresch, B.; Viegas, S.; Cramer, B.; Humpf, H. U. Multi-mycotoxin analysis using dried blood spots and dried serum spots. *Analytical and Bioanalytical Chemistry* **2017**, *409*(13), 3369.

5. Xue, K. S.; Cai, W. J.; Tang, L. L.; Wang, J. S. Aflatoxin B-1-lysine adduct in dried blood spot samples of animals and humans. *Food and Chemical Toxicology* **2016**, *98*, 210.

6. Viegas, S.; Osteresch, B.; Almeida, A.; Cramer, B.; Humpf, H.-U.; Viegas, C. Enniatin B and ochratoxin A in the blood serum of workers from the waste management setting. *Mycotoxin Research* **2018**, *34*(2), 85.

7. Renaud, J. B.; Walsh, J. P.; Sumarah, M. W. Optimization of Aflatoxin B1-Lysine Analysis for Public Health Exposure Studies. *Toxins* **2022**, *14*(10), 672.

8. Vidal, A.; Belova, L.; Stove, C.; De Boevre, M.; De Saeger, S. Volumetric Absorptive Microsampling as an Alternative Tool for Biomonitoring of Multi-Mycotoxin Exposure in Resource-Limited Areas. *Toxins (Basel)* **2021**, *13*(5), 345.

9. Schmidt, J.; Cramer, B.; Turner, P. C.; Stoltzfus, R. J.; Humphrey, J. H.; Smith, L. E.; Humpf, H.-U. Determination of Urinary Mycotoxin Biomarkers Using a Sensitive Online Solid Phase Extraction-UHPLC-MS/MS Method. *Toxins* **2021**, *13*(6), 418.

10. Njumbe Ediage, E.; Diana Di Mavungu, J.; Song, S.; Wu, A.; Van Peteghem, C.; De Saeger, S. A direct assessment of mycotoxin biomarkers in human urine samples by liquid chromatography tandem mass spectrometry. *Analytica Chimica Acta* **2012**, *741*, 58.

11. Solfrizzo, M.; Gambacorta, L.; Lattanzio, V. M. T.; Powers, S.; Visconti, A. Simultaneous LC–MS/MS determination of aflatoxin M1, ochratoxin A, deoxynivalenol, de-epoxydeoxynivalenol, α and β-zearalenols and fumonisin B1 in

urine as a multi-biomarker method to assess exposure to mycotoxins. *Analytical and Bioanalytical Chemistry* **2011**, *401*(9), 2831.

12. Fan, K.; Guo, W.; Huang, Q.; Meng, J.; Yao, Q.; Nie, D.; Han, Z.; Zhao, Z. Assessment of Human Exposure to Five Alternaria Mycotoxins in China by Biomonitoring Approach. *Toxins* **2021**, *13*(11), 762.

13. Leroy, J. L.; Wang, J.-S.; Jones, K. Serum aflatoxin B1-lysine adduct level in adult women from Eastern Province in Kenya depends on household socio-economic status: A cross sectional study. *Social Science & Medicine* **2015**, *146*, 104.

14. Becker, S.; Kinny-Köster, B.; Bartels, M.; Scholz, M.; Seehofer, D.; Berg, T.; Engelmann, C.; Thiery, J.; Ceglarek, U.; Kaiser, T. Low sphingosine-1-phosphate plasma levels are predictive for increased mortality in patients with liver cirrhosis. *PLoS One* **2017**, *12*(3), e0174424.
